# *Andrographis paniculata* (Burm.f.) Nees Alleviates Methotrexate-Induced Hepatotoxicity in Wistar Albino Rats

**DOI:** 10.3390/life13051173

**Published:** 2023-05-12

**Authors:** Manisha Parthasarathy, Sabina Evan Prince

**Affiliations:** Department of Biotechnology, School of Bio Sciences and Technology, Vellore Institute of Technology, Vellore 632014, India

**Keywords:** *Andrographis paniculata*, methotrexate, drug-induced hepatotoxicity, antioxidant, cytokines, apoptosis

## Abstract

*Andrographis paniculata* is a herbal plant used in traditional medicinal approaches to treat various ailments and diseases. Methotrexate (MTX) is a clinically used immunosuppressant and anticancer drug. One of the increasing concerns with MTX use is liver toxicity. The aim of this study is to investigate the potential effect of aqueous leaf extract of *Andrographis paniculata* against methotrexate-induced hepatotoxicity. Wistar albino rats were grouped into five groups, and the drugs were administered. MTX (20 mg/kg b.w.) was intraperitoneally injected into rats on the ninth day alone. Aqueous leaf extract of *Andrographis paniculata* (500 mg/kg b.w./day) was orally administered for 10 days. We confirmed the beneficial effect of aqueous extracts of *Andrographis paniculata* on restoring the hepatic enzyme markers, lipid profile, antioxidant level, anti-inflammatory marker (IL-10), anti-apoptosis (bcl-2), significant suppression of inflammatory cytokines (TNF-α, and IL-6), apoptosis marker (caspase 3) and cellular tissue damage caused by MTX. Overall, we revealed that *Andrographis paniculata* reduces critical aspects of oxidative stress, inflammatory processes, and apoptosis, thus protecting against methotrexate-induced hepatotoxicity.

## 1. Introduction

It is already well known that the liver has a prominent role in xenobiotic metabolisms so is highly susceptible to toxic threats. Drug-induced toxicity is a primary side effect that can lead to therapeutic limitations and hindrances in drug development. Hepatotoxicity caused by drugs is the main reason for the retraction of synthetic drugs from the market [1]. Hepatotoxicity has two mechanisms: intrinsic, which is dependent on the amount of the drug, and idiosyncratic, which is more unpredictable [2].

Methotrexate (MTX) is a widely known antifolate drug that inhibits the dihydrofolate reductase enzyme, which further inhibits the thymidylate synthesis pathway, eventually preventing DNA synthesis, the repair mechanism, and replication process [3]. Therefore, MTX is a clinically beneficial antimetabolite chemotherapeutic agent that is an effective therapeutic alternative commonly prescribed by physicians to treat cancer, ectopic pregnancy, and autoimmune diseases [4,5]. MTX treatment at a low dose of 2.5 mg and a high dose of 5 g is prescribed clinically [6,7]. However, long-term intake of MTX was reported to have severe toxicity, which was prominently found in the liver [8]. MTX, especially at a relatively high dosage, can increase the probability of cytopenias, infectious disease, and hepatotoxicity [9]. In the liver, MTX will be metabolized to form a polyglutamate, accumulating in the hepatocytes, increasing the risk of hepatotoxicity [10]. Several research studies have shown that oxidative stress plays a role in the mechanism of MTX-induced hepatic injury [11]. MTX-induced hepatotoxicity is caused by the development of reactive oxygen species (ROS) production, which ultimately reduces the potential activity of antioxidants and results in cell apoptosis and organ injury [12]. Furthermore, it has been demonstrated that TNF-α is crucial for maintaining liver equilibrium and links inflammation to apoptosis. According to previous studies, elevated levels of TNF-α, IL-6, and other cytokines trigger apoptotic pathways resulting in methotrexate-induced hepatotoxicity [13,14]. MTX is reported to cause hepatotoxicity when used for a prolonged period, even at a minimal dosage [15].

*Andrographis paniculata* (Burm.f.) Nees (Family: *Acanthaceae*) is a herbal plant naturally found in subtropical countries such as India (Kalmegh), China (Chuan-Xin-Lian), Vietnam (Xuyên tâm liên), Malaysia (Hempudu Bumi), Japan (Senshinren) and Thailand (Fah Tha Lai) and in Scandinavia (green chiretta) [16]. Because of its strong bitter flavour, *Andrographis paniculata* is acknowledged as the “King of Bitters” and is used as a bitter tonic [17]. *Andrographis paniculata* plant extracts showed the uppermost flavonoids, tannins, alkaloids, polyphenols, and triterpenoids through phytochemical analysis. This plant has antioxidant, anti-inflammatory, and antibacterial properties and antihyperglycemic effects [18]. It is ethnobotanically used for treating snake bites, scabies, bug bites, skin eruptions, gonorrhoea, bronchitis, malaria, jaundice, diabetes, leprosy, dysentery, anthelmintic, fever, cardiac diseases, and hepatic diseases [19]. Therefore, *Andrographis paniculata* may provide an alternative or adjuvant therapy to protect against drug-induced hepatotoxicity.

Silymarin (SLY) is a polyphenolic flavonoid extracted from the fruit and seeds of *Sylibum marianum,* commonly known as milk thistle [20]. It is frequently employed for the treatment of hepatic and gallbladder ailments. SLY exerts its pharmacological effects by modulating or suppressing β-glycoprotein, cell transporters, and estrogenic and nuclear receptors, which offer protection against drug-induced toxicity [21]. Therefore, we have employed SLY as a reference drug in this study.

There have been no scientific reports on the protective properties of the aqueous leaf extract of *Andrographis paniculata* against hepatotoxicity induced by MTX, specifically regarding its anti-inflammatory and anti-apoptotic mechanisms. In the current study, we evaluated the potential activity of the aqueous leaf extract of *Andrographis paniculata* against MTX-induced hepatotoxicity, focusing on biochemical assays, pro and anti-inflammatory cytokines, and apoptotic and anti-apoptotic protein expression levels.

## 2. Materials and Methods

### 2.1. Drugs and Chemical Reagents

MTX injection (folitrax) was purchased from IPAC Laboratories Ltd., Maharashtra, India. Commercially available silymarin (SLY) tablets were acquired from Micro Labs Pvt. Ltd. of Solan, Himachal Pradesh, India. Commercially accessible diagnostic kits for biochemical analysis were bought from Span Diagnostics Ltd. of Surat, Gujarat, India. Antioxidant parameter levels for the liver were analysed using standard protocols. TNF-α, IL-6, and IL-10 ELISA kits were purchased from Sigma-Aldrich of Bangalore, Karnataka, India. Caspase-3 and BCL-2 antibodies were obtained from Taurus Inc in India for Western blotting analysis.

### 2.2. Plant Extraction

Initially, “The Plant List” (http://www.theplantlist.org) and “Royal Botanic Gardens, Kew/Missouri Botanical Garden” (http://mpns.kew.org/mpns-portal/) confirmed the current plant species and the scientific name, and the date of access is 4 July 2022. *Andrographis paniculata* leaf powder (batch number: J130) is commercially available and was purchased from Jeyam Herbals, Madurai, Tamil Nadu, India. In the distilled water, the leaf powder was mixed and incubated with continuous shaking for 24 h using an electric shaker. The mixture was suction-filtered under the built-in vacuum pressure of a Buchner funnel and grade 2 (8 µm) Whatman filter paper. The filtrate sample was vacuum-concentrated in a rotary evaporator (Lork), and it was lyophilized and stored at −80 °C until the yield was used. The extract dosage was fixed based on a literature survey [22,23].

### 2.3. Gas Chromatography–Mass Spectroscopy Analysis of Andrographis paniculata

The *Andrographis paniculata* leaf extract was analysed for its chemical composition using gas chromatography–mass spectrometry (GC-MS) (make: Perkin Elmer, GC model: Clarus 680 and MS model: Clarus 600 (EI)). The leaf sample was added to the GC column, and the temperature was programmed to start at 60 °C for 2.5 min, ramping up at 10 °C/min to 300 °C and held for 6 min. The mass detector conditions consisted of a transfer line temperature of 240 °C, an ion source temperature of 240 °C, and an electron impact ionization mode at 70 eV, with a scan time of 0.2 s and a scanning interval of 0.1 s. The injector temperature was set to 260 °C. A 1 μL sample was loaded into the GC-MS and separated using an Elite-5 MS column (30.0 m, 0.25 nm ID, 250 μm df) with helium gas as a carrier at a column velocity flow of 1.0 mL/min to separate the components. The software TurboMass version 5.4.2 was utilised to retrieve the compounds from the library search. The obtained peaks were compared to the National Institute of Standards and Technology (NIST) research library’s mass spectral scan range of 40–600 (m/z) to determine the molecule.

### 2.4. Animals

Female Wistar albino rats (150–200 g) used in the current experimental study were acquired from the Animal House, VIT, Vellore, Tamil Nadu, India. The experimental approach was authorized by the VIT Institutional Animals Ethical Committee (Reg no: VIT/IAEC/18/Dec2020/16) under the appropriate guidelines of the Indian CPCSEA. Before the experiment started, the rats were acclimatized for a week. The rats were kept in a well-controlled room with a properly controlled temperature and light source. The pellet given to rats was purchased from Hindustan Lever Ltd. in Mumbai, India.

### 2.5. Experimental Design

Rats were divided into five groups of six rats for the 10-day study period as follows:

Group 1: Normal control—Control group (no intervention);
Group 2: Toxic control—MTX (20 mg/kg b.w., i.p.) on the 9th day;
Group 3: Treated—Aqueous leaf extract of *Andrographis paniculata* (500 mg/ kg b.w./day, *p.o.*) for 10 days + single dose of MTX (20 mg/kg b.w., i.p.) on 9th day;
Group 4: Standard—SLY (100 mg/kg b.w., p.o.) for 10 days + single dose of methotrexate (20 mg/kg b.w., i.p.) on 9th day;
Group 5: Drug alone—Aqueous leaf extract of *Andrographis paniculata* (500 mg/kg b.w./day, p.o.) alone for 10 days.

The concentration of the drug for MTX [24], *Andrographis paniculata* [23], and SLY [25] and the treatment schedule [26] were fixed based on preliminary findings and a literature survey. MTX (folitrax-25) was purchased in a vial and administered intraperitoneally into the rats. The rats were euthanized after the final dose using ether anaesthesia. The blood from experimental rats was collected (approx. 2–4 mL/rat) through a cardiac puncture and centrifuged at 3000 rpm for 10 min to obtain serum. The acquired serum was used to evaluate the liver function markers. The liver was homogenized for antioxidant, lipid peroxidation, and cytokine evaluation and preserved with formalin fixation for histopathological and immunohistochemical processing.

### 2.6. Biochemical and Antioxidant Analysis

The hepatoprotective potential was evaluated using liver enzyme markers such as alanine aminotransferase (ALT), aspartate aminotransferase (AST), alkaline phosphatase (ALP), total cholesterol (TC), total and direct bilirubin, low-density lipoprotein (LDL), high-density lipoprotein (HDL), triglyceride albumin, total protein, and gamma-glutamyl transpeptidase (GGT).

Liver homogenized samples were used to estimate the antioxidant assays such as superoxide dismutase (SOD) [27], reduced glutathione (GSH) [28], catalase (CAT) [29], glutathione peroxidase (GPx) [30], and lipid peroxidation indicator malondialdehyde (MDA) [31].

### 2.7. Histopathological Analysis

The liver was excised from the experiment rats, and ice-cold phosphate-buffered saline (PBS) (0.1 M) was utilized to rinse impurities. A liver fraction was statically mounted in 10% formalin. Dehydration, clearance, and paraffin were used in the processing for histopathologic examination. Tissue portions were stained with haematoxylin and eosin (H&E), and a microscopic investigation of the architecture modifications of the tissues was carried out.

### 2.8. ELISA Assay

The liver homogenate samples stored at −20 °C were used to measure inflammatory (TNF-α and IL-6) and anti-inflammatory (IL-10) cytokines. These cytokines’ ranges were measured using the enzyme-linked immunosorbent assay (ELISA) kits ordered and purchased from Sigma-Aldrich, India. The procedure to evaluate the levels of TNF-α, IL-6, and IL-10 was as per the instructions provided by the manufacturer.

### 2.9. Immunohistochemical Staining

Immunohistochemistry analysis was performed on liver tissue samples to evaluate the protein expression degree of caspase-3 and Bcl-2. To inhibit the endogenous peroxidase activity, tissue sections were deparaffinized for 10 min with 3% H_2_O_2_. The liver tissue was heated at 121 °C with 10 mM citrate buffer for 30 min for the antigen extraction process. After cooling, the liver segments were treated with 5% BSA to deter non-specific protein binding. The sample tissue was incubated with primary antibodies such as caspase-3 and Bcl-2 overnight at 4 °C. After 3–4 extensive PBS washes, tissue sections were incubated for 20 min at 32 °C with a secondary biotin-conjugated antibody. Following this and an additional incubation with horseradish peroxidase (HRP)-labelled streptavidin, antibody binding was visualized by staining with diaminobenzidine (DAB) and lightly counterstaining with haematoxylin at room temperature for 10 s. Carl Zeiss microscopes attached to a digital camera were used to take images of the tissue sample.

### 2.10. Statistical Analysis

All the data represented in this study are mean ± standard error (n = 6). The statistical evaluation was executed via one-way ANOVA followed by a post hoc Tukey’s test to establish a significant difference between all five groups, and *p* < 0.05 was regarded as significant. GraphPad Prism version 8.0 was used for the graphical analysis, and data are discussed and presented in tables and graphs.

## 3. Results

### 3.1. Phytochemical Constituent Analysis of Andrographis paniculata through GC-MS

**Figure 1:**
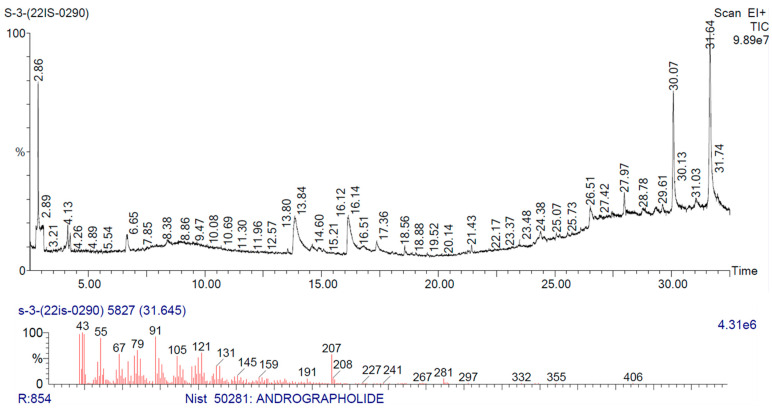
Chromatogram characterization acquired from GC-MS examination performed on *Andrographis paniculata* extract.

The GCMS analysis aimed to determine the phytochemicals contained in the samples. A chromatogram (Figure 1) was obtained, and the peaks were identified. The compounds from the peaks were discovered in the NIST library. GC-MS screening identified a majority of 15 compounds of the given plant extract. In Table 1, the common secondary data of the compounds are listed.

### 3.2. Protective Effect of Andrographis paniculata on Liver Enzyme Markers

**Figure 2:**
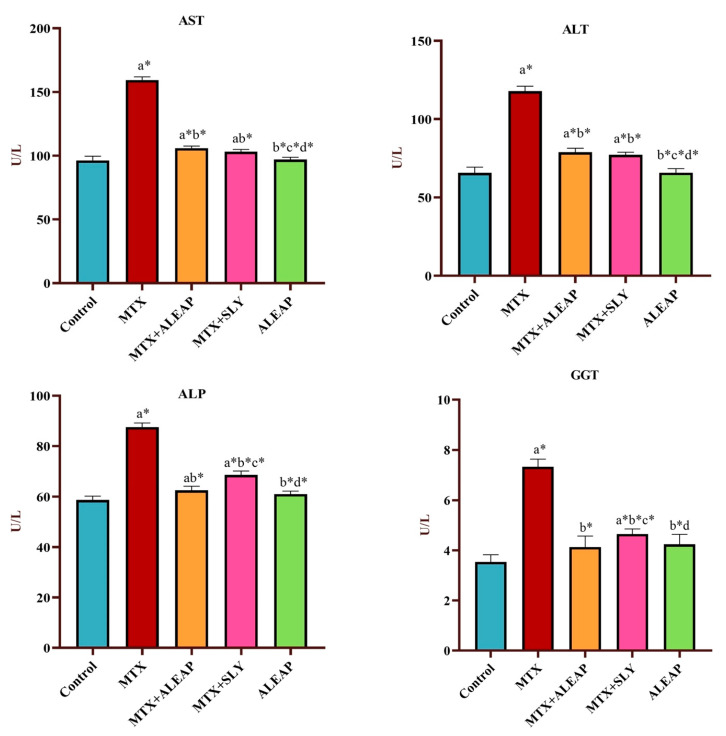
Protective effect of *Andrographis paniculata* on liver enzyme markers (AST, ALT, ALP, GGT) of MTX-induced rats. Each value shown is a mean ± SEM of N = 6 rats. The comparison between all five groups is as follows: a—control versus MTX, MTX + ALEAP, MTX + SLY, ALEAP; b—MTX versus MTX+ALEAP, MTX+SLY, ALEAP; c—MTX + ALEAP versus MTX + SLY, ALEAP; d—MTX + SLY versus ALEAP. The letters alone represent *p* < 0.05, and letters with * represent *p* < 0.001 (range of the statistical significance). The statistical data provided here were analysed by applying one-way ANOVA, subsequently followed by the Tukey post hoc test.

The effects of MTX toxicity on liver parameters were examined. As shown in Figure 2, MTX amplified the levels of ALT, ALP, AST, and GGT. Compared to the MTX rat group, administering *Andrographis paniculata* in combination with MTX prevented such an increase in liver parameters. The standard group SLY had reduced elevated liver markers almost similar to the treated group. In particular, there were no distinct changes in hepatic markers level between *Andrographis paniculata* alone and the control groups.

**Figure 3:**
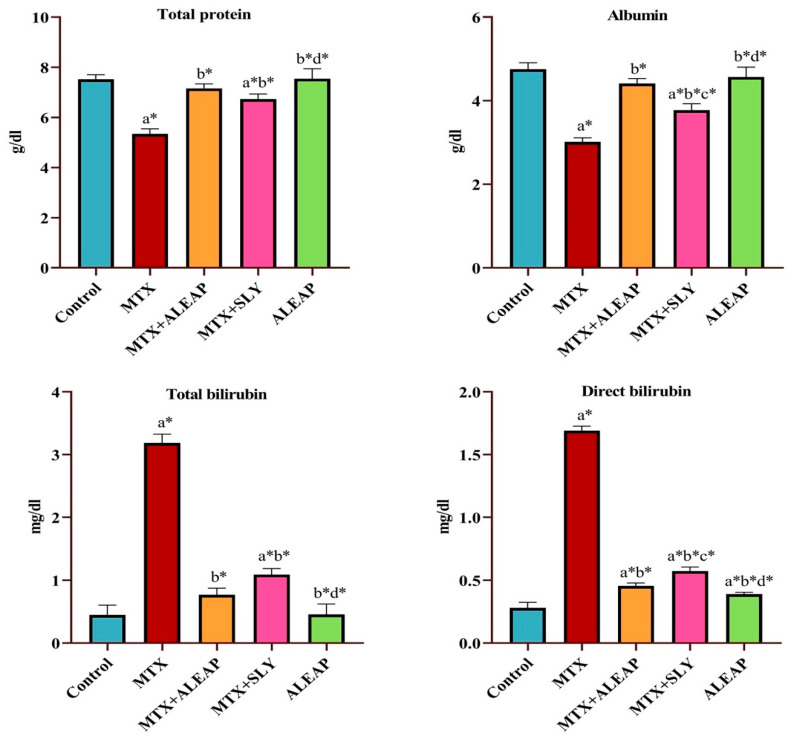
Protective effect of *Andrographis paniculata* on total protein, albumin, total bilirubin, and direct bilirubin of MTX-induced rats. Each value shown is a mean ± SEM of N = 6 rats. The comparison between all five groups is as follows: a—control versus MTX, MTX + ALEAP, MTX + SLY, ALEAP; b—MTX versus MTX + ALEAP, MTX + SLY, ALEAP; c—MTX + ALEAP versus MTX + SLY, ALEAP; d—MTX + SLY versus ALEAP. The letters alone represent *p* < 0.05, and letters with * represent *p* < 0.001 (range of the statistical significance). The statistical data provided here were analysed by applying one-way ANOVA, subsequently followed by the Tukey post hoc test.

In Figure 3, it can be seen that albumin and total protein levels were significantly depleted in the MTX-induced rat group. In contrast, total and direct bilirubin levels were elevated significantly (*p* < 0.05) in the MTX-administered rats. However, the treated group shows that these parameter changes were normalized almost to the control group. Compared to the SLY group, the *Andrographis paniculata*-treated group was far more successful in maintaining liver parameter concentration. There were no differences in the *Andrographis paniculata*-only treated group.

### 3.3. Protective Effect of Andrographis paniculata on Lipid Profile

**Figure 4:**
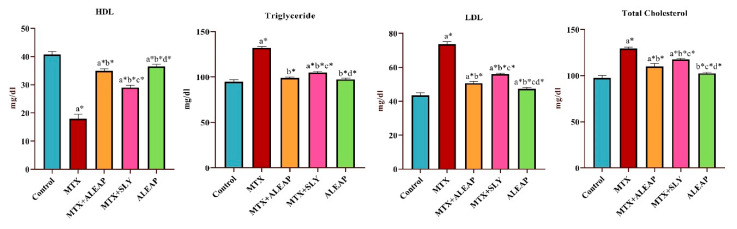
Protective effect of *Andrographis paniculata* on lipid profile (HDL, triglyceride, LDL, TC) of MTX-induced rats. Each value shown is a mean ± SEM of N = 6 rats. The comparison between all five groups is as follows: a—control versus MTX, MTX + ALEAP, MTX + SLY, ALEAP; b—MTX versus MTX + ALEAP, MTX + SLY, ALEAP; c—MTX + ALEAP versus MTX + SLY, ALEAP; d—MTX + SLY versus ALEAP. The letters alone represent *p* < 0.05, and letters with * represent *p* < 0.001 (range of the statistical significance). The statistical data provided here were analysed by applying one-way ANOVA, subsequently followed by the Tukey post hoc test.

In Figure 4, it can be seen that TC, LDL, and triglyceride levels were significantly higher in MTX-induced rats. Compared to normal rats, the HDL level of MTX significantly declined. The *Andrographis paniculata* and SLY treatments were significantly more potent in reinstating the levels of LDL, HDL, triglyceride, and TC. However, *Andrographis paniculata* is much more effective than the SLY. The lipid profile of rats that received *Andrographis paniculata* alone was normal.

### 3.4. Protective Effect of Andrographis paniculata on Antioxidant Biomarkers and Lipid Peroxidation

Table 2 shows the results on the protective role of *Andrographis paniculata* on MDA as an oxidative stress marker and SOD, CAT, GSH, and GPx as antioxidant enzymes. In Table 2, the outcomes show that MDA was significantly amplified (*p* < 0.05) in the MTX rats when compared to the normal rats (4.70 ± 0.02 for the MTX group vs. 2.18 ± 0.07 for the control). The co-administration of AP with MTX normalized the elevated level of MDA activity in the MTX group (2.85 ± 0.08 for MTX plus AP vs. 4.70 ± 0.02 for the MTX group).

In comparison with the control group, the MTX rats showed a significant reduction (*p* < 0.05) in the liver antioxidants such as SOD, CAT, GSH, and GPx (Table 2). Moreover, compared to the MTX-injected rats, *Andrographis paniculata* showed the ability to restore hepatic SOD, CAT, GSH, and GPx. There was a significant growth in antioxidant activity in the *Andrographis paniculata*-administered group. Compared to the corresponding control rats, the *Andrographis paniculata*-only group had no significant impact on the antioxidative markers.

### 3.5. Protective Effect of Andrographis paniculata on Liver Histopathology Alterations

**Figure 5:**
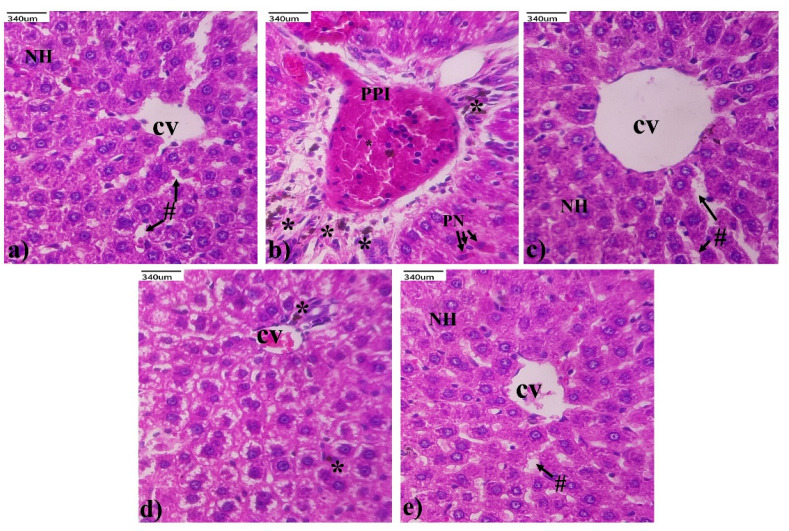
Protective effect of *Andrographis paniculata* on liver histopathology alteration of MTX-induced rats. Liver histopathology (H&E staining): Group (**a**) shows normal liver histocytes; Group (**b**) shows periportal inflammation, degenerative changes, portal vein congestion, Kupffer cell, and pyknotic nuclei; Group (**c**) shows changes in periportal inflammation, normal hepatocytes, and the central vein; Group (**d**) depicts mild inflammation; Group (**e**) shows normal hepatocyte nuclei and liver structure. Central vein (CV), Kupffer cell nucleus (*), sinusoidal spaces (#), periportal inflammation (PPI), and pyknotic nuclei (PN).

Figure 5 shows the histoarchitecture of the hepatocytes from the normal control, and the *Andrographis paniculata*-alone-treated group shows normal cellular details. However, the MTX-treated group shows periportal inflammation, degenerative changes, portal vein congestion, Kupffer cells, and pyknotic nuclei. By contrast, the pre-treatment of the *Andrographis paniculata*-treated group prevented the histopathological alterations caused by the MTX. Treatment with SLY alone attenuated the cellular damage by MTX. However, mild inflammation and very few Kupffer cells were observed.

### 3.6. Protective Effect of Andrographis paniculata on Anti- and Proinflammatory Cytokines through ELISA

**Figure 6:**
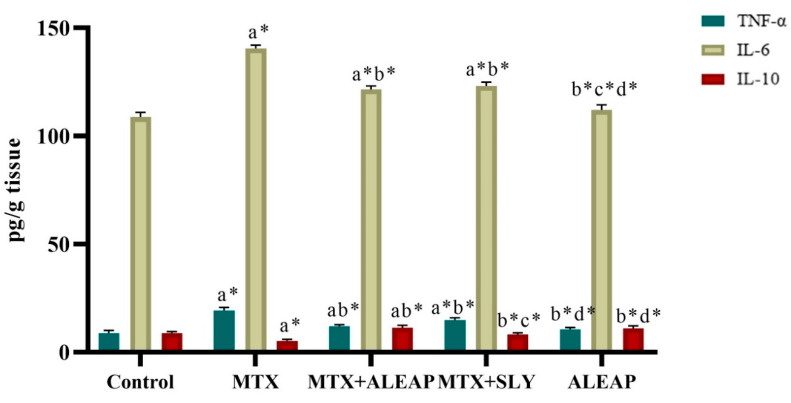
Protective effect of *Andrographis paniculata* on liver cytokines (TNF-α, IL-6, IL-10) of MTX-induced rats. Each value shown is a mean ± SEM of N = 6 rats. The comparison between all five groups is as follows: a—control versus MTX, MTX + ALEAP, MTX + SLY, ALEAP; b—MTX versus MTX + ALEAP, MTX + SLY, ALEAP; c—MTX + ALEAP versus MTX + SLY, ALEAP; d—MTX + SLY versus ALEAP. The letters alone represent *p* < 0.05, and letters with * represent *p* < 0.001 (range of the statistical significance). The statistical data provided here were analysed by applying one-way ANOVA, subsequently followed by the Tukey post hoc test.

Figure 6 shows that TNF-α and IL-6 were increased in MTX-induced rats compared to the control group. In contrast, IL-10 levels were decreased in the MTX-induced toxicity group. The *Andrographis paniculata*-treated group showed restoration of the TNF-α, IL-6, and IL-10 changes caused by MTX. The cytokine levels of IL-6 and TNF-α were reduced in the *Andrographis paniculata*-treated group, while IL-10 levels were higher than in the MTX groups. The SLY group had an almost similar outcome; however, *Andrographis paniculata* had a better effect on the cytokine level. The drug-alone group maintained a normal cytokine range, showing their beneficial effect.

### 3.7. Protective Effect of Andrographis paniculata on Protein Expression Degree of Caspase-3 and Bcl-2throughy Immunohistochemistry Analysis

**Figure 7:**
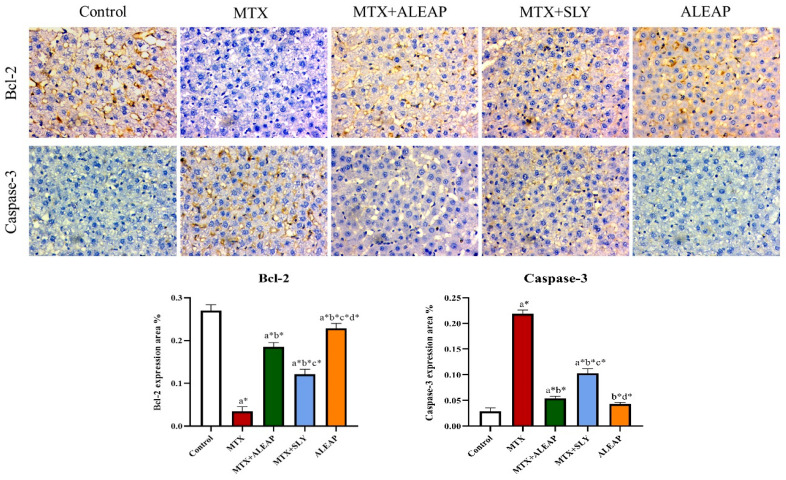
Protective effect of *Andrographis paniculata* on Caspase-3 and bcl-2 of MTX-induced rats. Each value shown is a mean ± SEM of N = 6 rats. The comparison between all five groups is as follows: a—control versus MTX, MTX + ALEAP, MTX + SLY, ALEAP; b—MTX versus MTX + ALEAP, MTX + SLY, ALEAP; c—MTX + ALEAP versus MTX + SLY, ALEAP; d—MTX + SLY versus ALEAP. The letters alone represent *p* < 0.05, and letters with * represent *p* < 0.001 (range of the statistical significance). The statistical data provided here were analysed by applying one-way ANOVA, subsequently followed by the Tukey post hoc test.

In Figure 7, it can be seen that the bcl-2 expression in MTX was nine-fold lower, and caspase-3 was higher than in normal rats. However, the *Andrographis paniculata*-treated MTX rats showed a reduced expression of caspase-3 and improved bcl-2 range. The SLY-treated group showed half the bcl-2 expression range and twice the caspase-3 of the control group. The *Andrographis paniculata*-alone-treated group maintained an almost similar range to the normal rats.

## 4. Discussion

Hepatotoxicity is caused by various factors such as physical, chemical, and biological factors resulting in hepatocyte degeneration, tissue damage, necrotic changes, and alteration in liver function [32]. MTX is a folate antagonist typically used as an antineoplastic, anti-inflammatory, and immunosuppressive agent, primarily in treating leukaemia, solid tumours, psoriasis, inflammatory conditions, and rheumatoid arthritis [33]. However, the most severe side effect of using MTX for long-term treatment results in hepatotoxicity caused by the accumulation of the principal metabolite 7-hydroxymethotrexate [34,35]. The MTX metabolite is deposited in hepatocytes, resulting in a prolonged intracellular existence in the tissue and, as a direct consequence, the initiation of oxidative stress and inflammatory damage takes place [36,37]. The hepatotoxicity of MTX has a significant impact on the clinical therapeutic benefits of MTX. Therefore, it requires effective additional herbal therapy. This study evaluated the protective effect of aqueous leaf extract of *Andrographis paniculata* against MTX-induced hepatotoxicity. The existence of multiple phytochemical constituents confirmed through GC-MS could be the primary source of bioactivity of the plant extracts. These compounds may be together or might separately contribute to their biological properties.

Injuries to the liver alter hepatocyte transport function and the permeability of the membrane, resulting in the leakage of cellular enzymes into the blood circulation. In this study, after MTX (20 mg/kg) administration, rats experienced significant changes in liver enzyme markers. MTX intoxication increased the serum concentration of ALP, AST, ALT, GGT, and total and direct bilirubin levels while decreasing total protein and albumin concentrations, indicating liver injury and impaired hepatic function. A previous study reported similar results in MTX-intoxicated rats [38,39]. However, pre-treatment with *Andrographis paniculata* normalized the altered enzyme markers, whereas it improved the total protein and albumin level, indicating an attenuation of MTX-induced toxicity. The liver has a fundamental role in lipids’ synthesis, transportation, and metabolism. Therefore, individuals with severe hepatic dysfunction would have an altered lipid profile [40]. HDL is recognized as good cholesterol because it acts as a scavenger in the bloodstream and removes LDL from the arteries by the reverse cholesterol transport pathway. As a result, in the current study, a low level of HDL and an increased level of LDL indicates hepatocyte damage [41]. Triglyceride is also known as bad cholesterol; excess retention of fat around artery walls may result in a heart attack [42]. Therefore, compared with the control group, the MTX-induced group shows increased levels of triglyceride and TC, indicating the toxic effect of MTX on lipid profile.

The mechanism of MTX-induced hepatic injury is obscure. However, the primary cause of MTX-related cellular injury is oxidative damage, including free radical production, lipid peroxidation, and pathological alterations [43]. The antioxidant defence mechanism eradicates the potentially toxic effects of free radicals in the liver. The activity of antioxidants was studied to evaluate the MTX-induced oxidative injury. SOD, CAT, GPx, and GSH are vital cellular antioxidants that guard the cell against generated ROS [44]. SOD was the first known free radical-scavenging enzyme that converts reactive superoxide oxygen to hydrogen peroxide via dismutation reactions. Catalase is a peroxisomal haemoprotein, further converting hydrogen peroxide to water and molecular oxygen, protecting the cell from ROS [45]. However, mitochondria lack catalase. Therefore, GPx is responsible for the reduction reaction of hydrogen peroxide to water, and suppresses lipid peroxides to their associated alcohols [46]. GSH is an antioxidant tripeptide comprising glutamate, glycine, and cysteine that is essential to defend mitochondria from xenobiotics toxicity, the generation of ROS, and regulating cell proliferation, metabolic activity, and apoptosis [47]. In our current study, the significant depletion of SOD, CAT, GSH, and GPx levels indicates MTX-induced oxidative damage.

On the other hand, lipid peroxidation MDA levels increased when compared to the normal control rats. MDA is a harmful substance, a key indicator for measuring membrane damage and lipid peroxidation level, as it is an end product of polyunsaturated fatty acid peroxidation [48,49]. In contrast, the administration of *Andrographis paniculata* has the potential to be hepatoprotective by increasing antioxidant capacity. Our current study has a similar result to Kalantari et al.’s work [50,51].

The biochemical evaluation was further supported by the histopathological evidence demonstrating the structural alterations in the hepatic tissue of the MTX-induced rats. It was observed that MTX at a dose of 20 mg/kg induces lesions, the degeneration of hepatocytes, periportal inflammation, portal vein congestion, Kupfer cells, and pyknotic nuclei in the liver. Previous studies have shown that these are standard representations of liver injury [52]. Pre-treatment with *Andrographis paniculata* resulted in significant improvements in the evaluated histopathologic parameters.

Cytokines are proteins with a low molecular weight released by various cell types, and have a prominent effect on cell communication [53]. Inflammatory cytokine production plays a key role in initiating hepatic tissue injury during drug-induced hepatotoxicity [54]. In our study, the MTX-induced toxicity group showed the production of proinflammatory cytokines (TNF-α and IL-6) and a reduction in anti-inflammatory cytokines (IL-10). Compared to the control group, the significant increase in inflammatory cell infiltration of the hepatocytes of the toxicity group indicates that MTX has a toxic effect on the liver. These changes were normalized after administering *Andrographis paniculata*, demonstrating its protective properties.

Free radical production further leads to cellular destruction, DNA damage, mitochondrial alteration, cytoskeletal protein degradation, and apoptosis. MTX can initiate oxidative stress, which accelerates apoptosis [55]. Caspase genes (caspase-3) are crucial in sustaining homeostasis by regulating cell apoptosis and inflammation [56]. In our study, immunohistochemistry analysis showed an elevated level of caspase 3 in liver tissue observed in the MTX group, showing their toxicity effect. On the other hand, a reduction in Bcl-2 protein (anti-apoptosis protein) expression level exhibits decreased anti-apoptosis activity. However, *Andrographis paniculata* treatment normalized the protein expression level of caspase-3 and Bcl-2. The SLY- and *Andrographis paniculata*-alone-treated rats demonstrated normal protein expression ranges in immunohistochemistry. *Andrographis paniculate* showed its protective effect against MTX-induced hepatotoxicity. A previous study indicated similar results in MTX-induced hepatotoxicity [55].

A limitation of our study is that we only evaluated the molecular changes in proteins related to methotrexate-induced hepatotoxicity. To provide a more comprehensive understanding of the pathway involved, it would be beneficial to perform gene expression analysis. Additionally, we faced limitations in the process of using aqueous *Andrographis paniculata* extract in GC-MS. While we could identify the main active ingredient in our methanol extract, we could not provide the same level of detail for the aqueous leaf extract of *Andrographis paniculata*. Future studies may benefit from validating the relevant library similarity of the aqueous extract in GC-MS analysis with reduced noise and increased accuracy.

## 5. Conclusions

In summary, the current study result concluded that pre-treatment with *Andrographis paniculata* in MTX-intoxicated rats has hepatoprotective activity through its ability to enhance antioxidants, normalize liver enzyme markers, reduce proinflammatory cytokines (TNF-α and IL-6), improve the anti-inflammatory (IL-10) and the apoptotic markers (caspase-3), and attenuate and enhance anti-apoptosis (Bcl-2). Further investigation can be carried out to understand the exact molecular mechanism.

## Figures and Tables

**Table 1:** The list of compounds present in *Andrographis paniculata* leaf extract according to the GCMS study.

S. no	RT (min)	Phytochemical Name	Molecular Formula	Molecular Weight	Peak Area (%)
1	2.518	Cyclobutanol	C_4_H_8_O	72	6.331
2	2.859	Acetic acid, 1-methylethyl ester	C_5_H_10_O_2_	102	19.949
3	4.129	3-methoxycarbonyl-3-methyl-1,2,4-trioxolane	C_5_H_8_O_5_	148	1.578
4	6.685	Oxalic acid, isohexyl pentyl ester	C_13_H_24_O_4_	244	2.593
5	13.843	1-octanamine, n-methyl-n-nitroso-	C_9_H_20_ON_2_	172	14.451
6	16.144	Heptanal	C_7_H_14_O	114	10.492
7	17.359	3,4-furandiol, tetrahydro-, cis-	C_4_H_8_O_3_	104	1.333
8	24.392	2-methyl-6-methylene-octa-1,7-dien-3-ol	C_10_H_16_O	152	0.859
9	26.513	1-tridecyne	C_13_H_24_	180	3.572
10	26.698	1,1-dodecanediol, diacetate	C_16_H_30_O_4_	286	0.954
11	27.973	2,6-lutidine 3,5-dichloro-4-dodecylthio-	C_19_H_31_NC_l2_S	375	2.070
12	29.334	1-propanamine, n,2-dimethyl-n-nitroso-	C_5_H_12_ON_2_	116	1.408
13	29.609	(2s,3s)-(-)-3-propyloxiranemethanol	C_6_H_12_O_2_	116	1.150
14	30.074	3,4-nonadien-6-yne, 5-ethyl-3-methyl-	C_12_H_18_	162	11.150
15	31.645	Andrographolide	C_20_H_30_O_5_	350	22.111

RT—retention time.

**Table 2:** Protective effect of *Andrographis paniculata* on liver antioxidant parameters of MTX-induced rats.

PARAMETERS	SOD (Units/min/mg Protein)	CAT (Units/min/mg Protein)	GSH (nmol/mg Protein)	GPx (nmol/min/mg Protein)	MDA (nmol/g Tissue)
Control	97.01 ± 0.53	77.15 ± 0.62	48.84 ± 0.3	43.63 ± 0.03	2.18 ± 0.07
MTX	58.86 ± 0.39 a*	38.42 ± 0.31 a*	25.06 ± 0.29 a*	24.80 ± 0.12 a*	4.70 ± 0.02 a*
MTX + ALEAP	83.60 ± 0.33 a*b*	74.79 ± 0.07 a*b*	45.92 ± 0.46 ab*	40.91 ± 0.13 a*b*	2.85 ± 0.08 a*b*
MTX + SLY	78.53 ± 0.35 a*b*c*	68.78 ± 0.02 a*b*c*	42.01 ± 0.12 a*b*c*	38.68 ± 0.15 a*b*c*	3.19 ± 0.01 a*b*c*
ALEAP	81.20 ± 0.45 a*b*cd*	75.16 ± 0.45 b*c*d*	47.04 ± 0.28 b*d*	41.63 ± 0.02 a*b*d*	2.45 ± 0.02 ab*c*d*

Each value shown is a mean ± SEM of N = 6 rats. The comparison between all five groups is as follows: a—control versus MTX, MTX + ALEAP, MTX + SLY, ALEAP; b—MTX versus MTX+ALEAP, MTX+SLY, ALEAP; c—MTX + ALEAP versus MTX+SLY, ALEAP; d—MTX+SLY versus ALEAP. The letters alone represent *p* < 0.05, and letters with * represent *p* < 0.001 (range of the statistical significance). The statistical data provided here were analysed by applying one-way ANOVA, subsequently followed by the Tukey post hoc test. SOD—superoxide dismutase; CAT—catalase; GPx—glutathione peroxidase; GSH—reduced glutathione; MDA—malondialdehyde; MTX—methotrexate; ALEAP—aqueous leaf extract of *Andrographis paniculata*; SLY—silymarin.

## Data Availability

Data will be provided on reasonable request.
